# Isolated pores dissected from human two-pore channel 2 are functional

**DOI:** 10.1038/srep38426

**Published:** 2016-12-12

**Authors:** Christopher J. Penny, Taufiq Rahman, Altin Sula, Andrew J. Miles, B. A. Wallace, Sandip Patel

**Affiliations:** 1 Department of Cell and Developmental Biology, University College London, London, WC1E 6BT, UK; 2 Department of Pharmacology, University of Cambridge, Cambridge, CB2 1PD, UK; 3 Institute of Structural and Molecular Biology, Birkbeck College, University of London, London, WC1E 7HX, UK

## Abstract

Multi-domain voltage-gated ion channels appear to have evolved through sequential rounds of intragenic duplication from a primordial one-domain precursor. Whereas modularity within one-domain symmetrical channels is established, little is known about the roles of individual regions within more complex asymmetrical channels where the domains have undergone substantial divergence. Here we isolated and characterised both of the divergent pore regions from human TPC2, a two-domain channel that holds a key intermediate position in the evolution of voltage-gated ion channels. In HeLa cells, each pore localised to the ER and caused Ca^2+^ depletion, whereas an ER-targeted pore mutated at a residue that inactivates full-length TPC2 did not. Additionally, one of the pores expressed at high levels in *E. coli*. When purified, it formed a stable, folded tetramer. Liposomes reconstituted with the pore supported Ca^2+^ and Na^+^ uptake that was inhibited by known blockers of full-length channels. Computational modelling of the pore corroborated cationic permeability and drug interaction. Therefore, despite divergence, both pores are constitutively active in the absence of their partners and retain several properties of the wild-type pore. Such symmetrical ‘pore-only’ proteins derived from divergent channel domains may therefore provide tractable tools for probing the functional architecture of complex ion channels.

Voltage-gated ion channels selective for Ca^2+^ (Ca_V_), Na^+^ (Na_V_) and K^+^ (K_V_) perform a plethora of functions in both excitable and non-excitable cells. Mutations in these channels are the causal basis of numerous diseases, thereby rendering them clinically-relevant drug targets^1^. They are composed of four domains that form a central pore, with peripheral voltage sensors. Each domain consists of six transmembrane helices comprising the voltage sensor (S1-S4) and pore (S5-S6) regions. In K_V_ and prokaryotic Na_V_, the domains are separate subunits that form a tetramer, resulting in symmetrical pores. In contrast, eukaryotic Ca_V_ and Na_V_ are single polypeptide chains with four divergent domains, giving rise to asymmetric pores^1^,^2^. This architectural similarity suggests an evolutionary trajectory whereby a primordial gene encoding a one-domain channel underwent two rounds of intragenic duplication and divergence to generate the extant four-domain channels (Fig. 1A)^3^,^4^.

Two-pore channels (TPCs) are less well characterised members of the voltage-gated ion channel superfamily that, unusually, localise to intracellular acidic Ca^2+^ stores^5^. In animals, they are activated by the second messenger NAADP to release Ca^2+^ from the endo-lysosomal system, and are an important part of the cellular signalling apparatus^6^,^7^,^8^. Furthermore, TPCs are rapidly emerging as potential therapeutic targets^9^,^10^,^11^. Recent crystal structures of a plant TPC^12^,^13^,^14^ have confirmed earlier biochemical reports that they form dimers from two-domain (DI and DII) subunits^15^,^16^. This structural organisation identifies TPCs as a key intermediate in the evolution of voltage-gated ion channels from one-domain to four-domain channels (Fig. 1A). Indeed, phylogenetic analyses of the individual TPC domains supports this conclusion, indicating that they are substantially diverged from one another, and are instead more related to equivalent domains in four-domain channels^17^.

The modularity of the pore regions in symmetrical (often prokaryotic) channels is established^18^,^19^,^20^,^21^. For example, the isolated pore of a Na_V_ from a marine bacterium forms an open, folded tetramer that is constitutively active, thereby supporting Na^+^ flux in the absence of the voltage sensor^22^. Similar results have been found for ‘pore-only’ proteins derived from other prokaryotic channels^18^,^20^,^23^. The functional architecture of asymmetric ion channel pores, however, is less clear due to the inherent difficulties in analysing larger, more complex, multi-domain proteins. Here, we examined whether protein dissection could be applied to the two divergent pore regions of TPCs as a means to probe the properties of asymmetric multi-domain proteins.

## Results

### Design of TPC2 pore-only proteins

**Figure 1:**
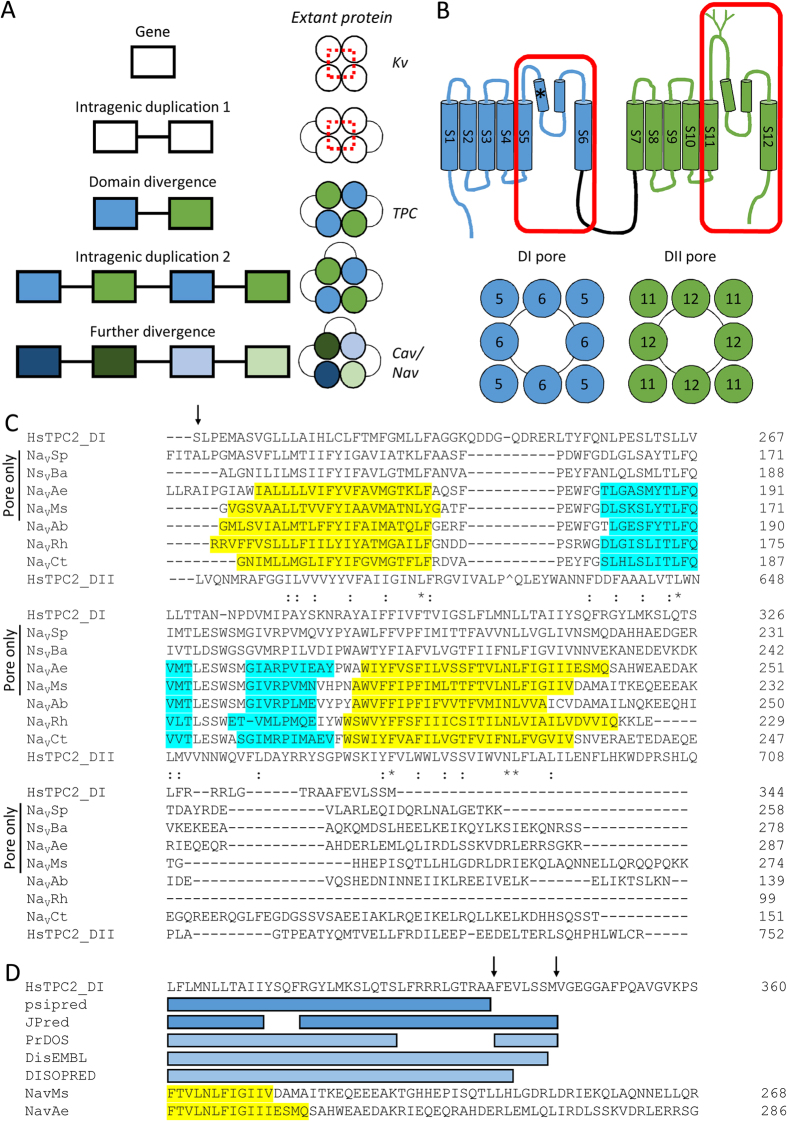
Design of TPC2 pore-only proteins. (**A**) Proposed trajectory of evolution for one-, two- and four-domain voltage-gated ion channels. Dashed box highlights the ancestral pore. (**B**) Schematic of human TPC2 highlighting the two pore regions (red boxes), a residue required for channel activity (*) and *N*-glycosylation sites (top), and predicted assembly of isolated pore-only proteins derived from each domain (bottom). (**C**) Multiple sequence alignment of the pore regions of human TPC2 (HsTPC2) domain I (DI) and domain II (DII) pores with the previously characterised pore-only bacterial channels (Na_V_Sp, Ns_V_Ba, Na_V_Ae and Na_V_Ms) along with the pore regions of the structurally-resolved full length Na^+^ channels (Na_V_Ab, Na_V_Rh and Na_V_Ct). For channels or pores where the structure has been solved, the transmembrane helices and re-entrant pore helices are highlighted in yellow and cyan, respectively. For HsTPC2 DII, a short turret loop was removed, denoted by ^. (**D**) Predicted helical (dark blue) or ordered (light blue) sequences at the putative end of S6 of DI from human TPC2. Arrows indicate the last residues of the two DI pore constructs (M344 for the HeLa expression, and A337 for *E. coli* expression). For comparison, the sequences of the C-termini of Na_V_Ms and Na_V_Ae are shown, with the ends of S6 highlighted.

We designed constructs encoding each of the pore regions of human TPC2 (S5-S6 and S11-S12) (Fig. 1B). To define the pore boundaries, a multiple sequence alignment was performed with the pore regions of previously characterised pore-only and full-length bacterial Na^+^ channels (Fig. 1C). The N-termini of TPC pores were chosen as midway between the N-termini of pore-only Na^+^ channel constructs, just upstream of the S5 regions (Fig. 1C, arrow). However, the C-terminus of TPC2 possesses little sequence similarity with these channels (Fig. 1C). For DI, we addressed this by performing secondary structure predictions which suggested an extended S6 helix (Fig. 1D). Additional disorder predictions indicated where regular secondary structure declined, thereby identifying a suitable region for truncation (Fig. 1D, arrows). For DII, we included the entire C-terminus. From these analyses, the pore regions for TPC2 were defined to be residues S212-M344 (DI pore) and L575-R752 (DII pore).

### Expression of TPC2 pore-only proteins

We generated tagged constructs corresponding to each pore and tested their expression in both a homologous human cell line (HeLa) for cell biology studies and a heterologous system (*E. coli*) for biochemical and structural analyses.

**Figure 2:**
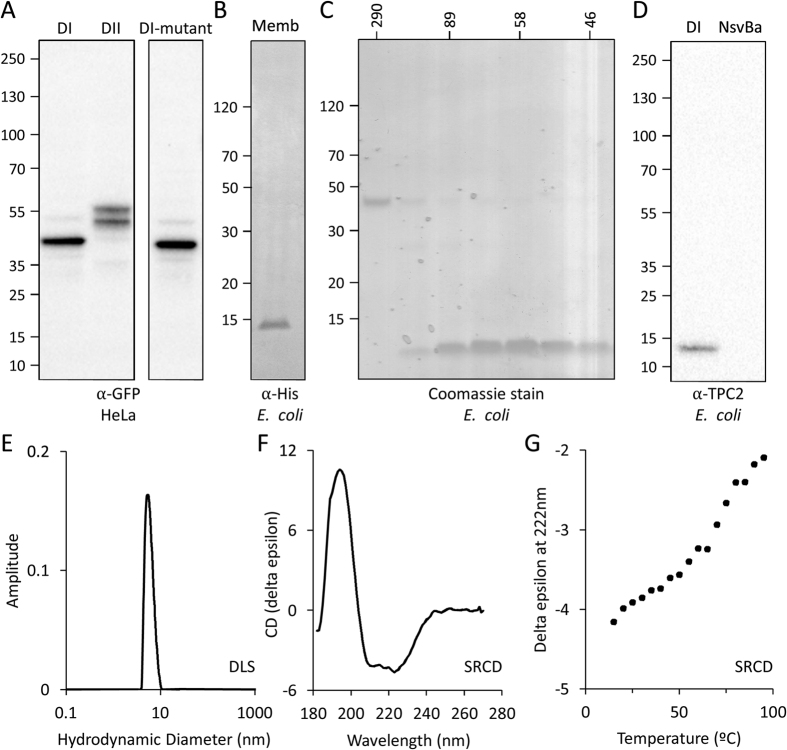
Expression of TPC2 pore-only proteins. (**A,B**) Western blots against pores expressed in HeLa (**A**) and *E. coli* (**B**) homogenates. Expected sizes: 42 kDa for DI pore-GFP, 48 kDa for DII pore-GFP, 16 kDa for hexaHis-DI pore. (**C**) SDS PAGE of gel filtration fractions obtained during purification of TPC2 DI pore with molecular weight calibration standards displayed above. (**D**) Western blot using a α-TPC2 antibody of purified TPC2 DI and Ns_V_Ba pores (expected sizes: 16 kDa for TPC2 and 15 kDa for Ns_V_Ba). (**E**) Mass distribution of the purified pore using dynamic light scattering. (**F**) SRCD spectrum of purified TPC2 DI pore. (**G**) Thermal denaturation of purified TPC2 DI pore monitored using SRCD spectroscopy at 222 nm.

Both TPC2 pores tagged with GFP expressed readily in Hela cells (Fig. 2A). The DII pore was resolved as a doublet, likely reflecting full and core *N*-glycosylation of the intraluminal loop between S11 and the first pore helix. These data suggest that the pore undergoes appropriate post-translational modification similar to the full length channel^24^. We also successfully expressed a mutant DI pore (Fig. 2A) equivalent to the inactive full length channel (L265P) (Fig. 1B, asterisk)^25^.

Large quantities of pure protein are required for biophysical and structural studies. *E. coli* are a useful expression system in which to achieve this, however this is notoriously challenging for human constructs, particularly those encoding membrane proteins. In initial trials, we partially purified a His-tagged DI pore construct that included an extended C-terminus, corresponding to the inter-domain loop. This was achieved through varying the temperature and time of protein induction, the detergent used for solubilisation and the matrix for affinity purification (Fig. S1A–C). Based on these findings and bioinformatics analyses of the C-terminal end of the DI pore (Fig. 1D), we similarly tested a series of truncated constructs (data not shown). This culminated in the efficient expression of a shortened construct corresponding to residues S212-A337 (Fig. S1D) that was effectively solubilised with DM and purified by sequential cobalt-based affinity and size exclusion chromatography (Fig. 2B,C). The identity of the protein was confirmed by Western blotting using an anti-TPC2 antibody (Fig. 2D), and by mass spectrometry (data not shown).

The elution volume of the purified protein during size exclusion chromatography indicated an apparent molecular weight of ~60 kDa (Fig. 2C), consistent with a tetrameric assembly (predicted size of monomer = 15 kDa). However, the absence of tryptophan residues precluded more accurate quantitation using UV absorbance. We therefore used dynamic light scattering to estimate particle size, which indicated a single species (39% polydispersity) with a hydrodynamic diameter of 6.1 nm (Fig. 2E), equivalent to the dimensions of a pore-only tetramer^22^. To probe the secondary structure of the protein, we used synchrotron radiation circular dichroism spectroscopy (SCRD), an advanced technique that enables detailed characterisation of proteins in detergent-containing buffers^26^. This demonstrated a well-ordered structure comprising 59 ± 3% helix (Fig. 2F), very similar to the 58% helical content expected from the secondary structure prediction. Furthermore, we measured a significant and cooperative loss of helical secondary structure upon thermal melting, similar to that seen for tetrameric Na^+^ channel pores^19^,^20^,^27^ (Fig. 2G) suggesting the purified protein assembles into a stable and well-folded oligomer. Together, these data demonstrate that both pores of human TPC2 can be expressed in isolation to form stable homo-tetrameric complexes that are correctly folded and processed.

### Functionality of TPC2 pore-only proteins

**Figure 3:**
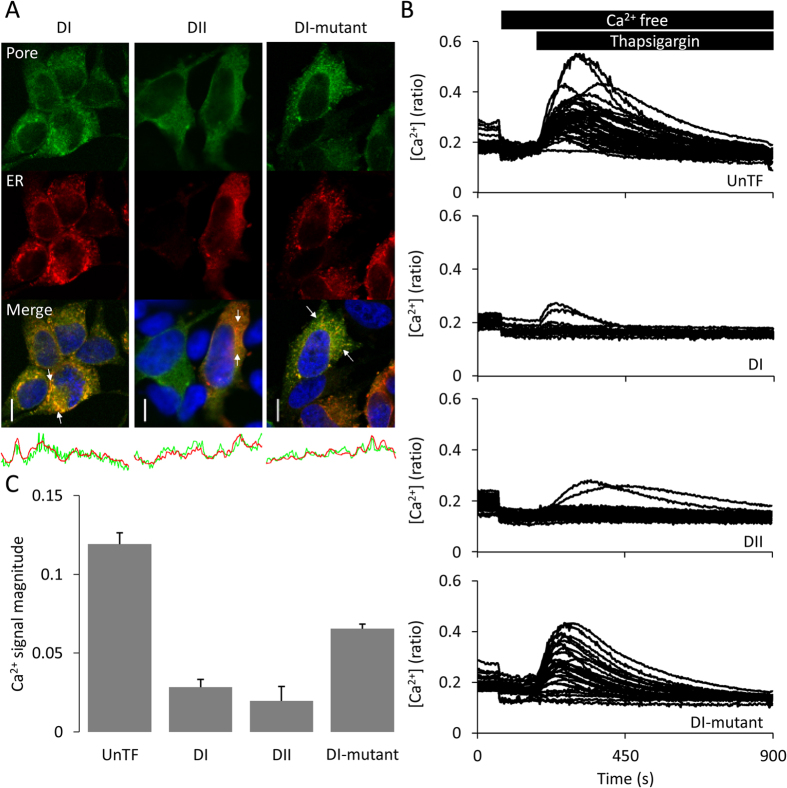
Functionality of TPC2 pore-only proteins in live cells. (**A**) Confocal images of HeLa cells co-expressing an ER marker (DsRed2-ER, red) and the indicated GFP-tagged pore (green). Nuclei (blue) were stained with DAPI. Scale bar 10 μm. Co-localisation calculated between arrowheads (below). (**B**) Cytosolic Ca^2+^ levels of individual HeLa cells expressing the indicated pore-only construct, stimulated with 1 μM thapsigargin in the absence of extracellular Ca^2+^. (**C**) Bar graphs quantifying signal magnitudes (*n* = 3–4).

Next, we assessed the activity of the pores. Confocal microscopy revealed co-localisation of the pores with an ER (Fig. 3A) but not a lysosomal (Fig. S2) marker in HeLa cells. Because TPCs are Ca^2+^-permeable^5^ and the ER is a large store of Ca^2+^, we reasoned that the pores would deplete ER Ca^2+^ levels if they are constitutively active, similar to prokaryotic pore-only proteins^18^,^19^,^20^,^22^. To test this, we estimated ER Ca^2+^ content by measuring Ca^2+^ leaks upon blocking Ca^2+^ uptake with thapsigargin. These cytosolic Ca^2+^ signals were substantially reduced in cells expressing either the DI or DII pore but not the mutant DI pore (Fig. 3B,C), consistent with constitutive activity.

**Figure 4:**
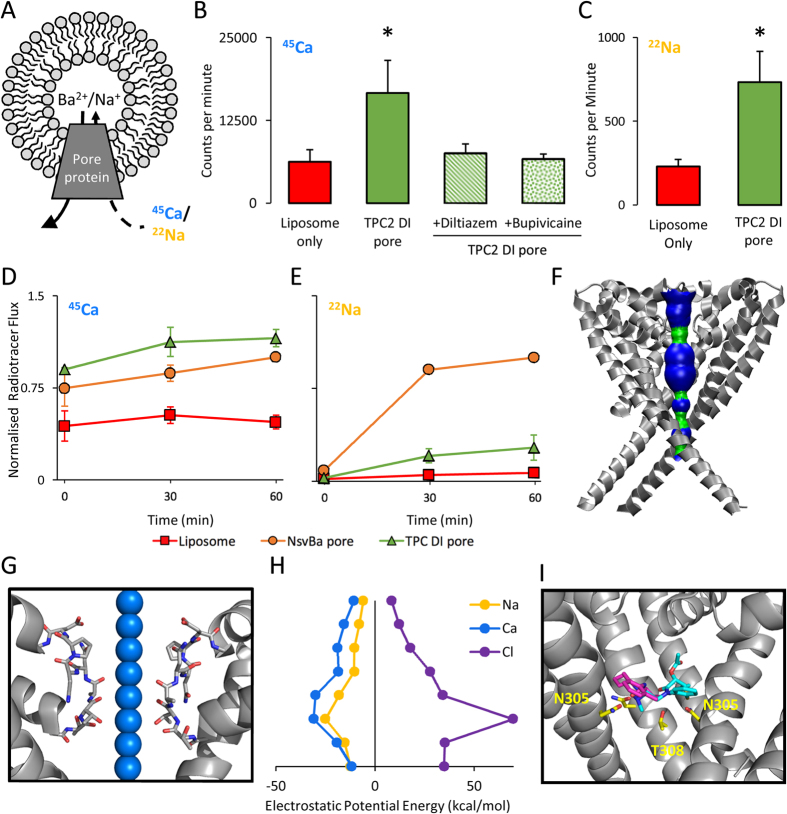
Functional reconstitution of a purified TPC2 pore-only protein. (**A**) Schematic depicting the radiotracer uptake method for assessing pore functionality. (**B,C**) Accumulation of ^45^Ca (**B**) or ^22^Na (**C**) following a 60 min incubation with control liposomes or liposomes incorporating the purified DI pore of TPC2 (*n* = 6–9; *represents p < 0.05, as assessed by unpaired Student’s *t*-test). Experiments were performed in the absence and presence of 100 μM diltiazem or 1 mM bupivacaine as indicated (*n* = 3–4). (**D,E**) Time course for uptake of ^45^Ca (**D**) or ^22^Na (**E**) by the TPC2 DI pore relative to pore of Ns_V_Ba. Data (*n* = 3–4) are normalised to the Ns_V_Ba signal at *t* = 60 minutes. (**F**) Homology model of DI pore (grey), displaying the solvent-accessible putative ion conduction pathway as a surface plot. Blue indicates diameters >5 Å, and green diameters between 2.3 and 5 Å. (**G,H**) Calculated electrostatic potential energies for Ca^2+^, Na^+^ and Cl^−^ (**H**) at the highlighted points throughout the selectivity filter of the DI pore (**G**). Blue spheres reflect the diameter of Ca^2+^ ions used within the calculation. (**I**) Zoomed view of diltiazem (cyan) and bupivacaine (magenta) docked to the structural model in (**F**), with a single monomer removed for clarity. Residues (full length numbering) predicted to form hydrogen bonds with the drugs are highlighted in yellow.

To independently measure pore functionality, we incorporated the purified DI pore into liposomes and measured radiotracer uptake (Fig. 4A). Uptake of ^45^Ca into reconstituted liposomes was significantly higher than liposomes without the pore (Fig. 4B). Additionally, diltiazem (a Ca_V_ blocker) and bupivacaine (a Na_V_ blocker) both reduced ^45^Ca uptake by the DI pore (Fig. 4B) but had little effect on pore-free liposomes (data not shown), thereby attesting to specificity.

Although several studies suggest that TPCs are non-selective cation channels^9^,^25^,^28^, others suggest they may be Na^+^-selective^29^. We therefore also examined liposomal ^22^Na uptake and found that the DI pore was able to support Na^+^ accumulation (Fig. 4C). Further experiments compared ^45^Ca and ^22^Na uptake relative to that of the isolated pore of Ns_V_Ba, a non-selective ion channel from *Bacillus alcalophilus*^30^,^31^. As shown in the time courses presented in (Fig. 4D,E), the Ns_V_Ba pore supported rapid uptake of ^45^Ca and slower uptake of ^22^Na. Parallel experiments with the TPC2 DI pore revealed comparable ^45^Ca uptake but substantially less ^22^Na uptake.

To rationalize these findings, we probed the properties of the DI pore computationally by generating a structural model using the plant TPC crystal structure as a template (Fig. 4F)^12^. A solvent-accessible cavity spanned the entire length of the putative ion conduction pathway (Fig. 4F), consistent with observed constitutive activity (Figs 3B,C and 4B,C). Moreover, electrostatics calculations indicated negative (*i.e.* favourable) potential energies for Ca^2+^ and Na^+^ but not Cl^−^ at intervals within the selectivity filter (Fig. 4G,H) in accord with cation permeability (Fig. 4B–E). We also performed molecular docking studies with diltazem and bupivacaine, which both bound within the pore cavity approximately midway between the selectivity filter and the bundle crossing (Fig. 4I). The predicted free energies of interaction were −6.6 and −5.6 kcal/mol, respectively, commensurate with drug block (Fig. 4B). *In silico analyses* thus support our experimental findings.

Collectively, we provide several lines of independent evidence to indicate that isolated TPC pore regions are capable of forming functional pores.

## Discussion

In this study, we have used a variety of complementary techniques to probe the functional architecture of an asymmetrical two-domain voltage-gated ion channel through analyses of each of the diverged pore regions of human TPC2.

Previous studies using isolated pores derived from prokaryotic K_V_^18^ and Na_V_^19^,^20^,^22^, which are symmetrical channels, have shown that they are constitutively active. We provide two independent lines of evidence here to indicate that both pores of human TPC2 are also active upon isolation. In the first approach, we expressed the pores in Hela cells and found that they localized to the ER (Fig. 3A). Such an intracellular location precludes standard electrophysiological analyses that require surface expression. However, we took advantage of the fact the ER is the major store of Ca^2+^ to show that expression of the wild-type pores, but not the mutant DI pore, resulted in ER Ca^2+^ depletion (Fig. 3B,C). Such an approach provides a novel means of inferring Ca^2+^ permeability of pores that fail to traffic to the plasma membrane.

In our second approach, we succeeded in expressing one of the pores in *E. coli* (Fig. 2B). This is not trivial for human membrane proteins. We focussed on the DI pore because it has a shorter intraluminal loop that lacks sites for N-glycosylation (Fig. 1B), a post translational modification not supported by *E. coli*. After much optimisation (Fig. S1), we purified sufficient quantities (Fig. 2C) to allow functional analyses which showed that the pore supported both Ca^2+^ and Na^+^ uptake upon reconstitution (Fig. 4). Ca^2+^ uptake was rapid, likely reflecting the presence of EGTA in the intra-liposomal solution, and comparable to the pore of Ns_V_Ba (Fig. 4D). Ns_V_Ba is non-selective channel with a *P*_*Ca*_*/P*_*Na*_ of ~1.5^30^ Because Na^+^ uptake was more modest for the DI pore relative to Ns_V_Ba (Fig. 4E), we speculate that the DI pore may be more permeable to Ca^2+^ than Na^+^, results supported by our electrostatics calculations (Fig. 4F–H). However, direct electrophysiological analysis is required to establish this. Such an estimate is inconsistent with marked Na^+^ selectivity reported for recombinant full-length TPC2 in some studies^29^, and is instead more comparable to that of endogenous TPC2 (*P*_*Ca*_*/P*_*Na*_ = 0.57–0.86)^28^.

The wild-type TPC pore is a dimer comprising alternating pore domains^12^,^13^,^14^. The domains of TPCs have undergone substantial divergence such that they appear to be more related to equivalent domains in four-domain channels than to one another^17^. Yet as shown here dissecting symmetrical pore-only proteins from *either* domain creates stable structures that retain essential elements of pore functionality. We liken homomeric TPC pores to ancestral TPCs formed upon intragenic duplication but prior to divergence (Fig. 1A). We speculate that the contribution of DII within the wild-type pore can be substituted by DI, as exemplified by both the structural integrity (Fig. 2E–G), functionality and drug block (Fig. 4B–E) of the DI pore-only protein. Equally, the contribution of the DI can substituted by DII as exemplified by the functionality of the DII pore (Fig. 3B,C), although further biophysical, biochemical and pharmacological characterisations will be required for confirmation. This suggests that there is a certain degree of functional and architectural ‘redundancy’ in the pore make-up of multi-domain voltage-gated ion channels *i.e* either pore domain can assemble and function without the other. This parallels the observed functionality of a chimeric K_V_ from *Drosophila melanogaster* in which the pore region (S5-S6) of the symmetrical *Shaker* channel is replaced with a pore region (M1-M2) of asymmetric KCNK0^32^. The latter is a member of the two-pore domain K^+^ family (K2P) that lack canonical voltage sensors but, like TPCs, have two pore domains^33^. The resulting symmetrical pore derived from an asymmetrical channel in the chimera (although not isolated) is thus conceptually similar to our TPC2 pore-only constructs.

Successful purification of a functional, stably folded, tetrameric TPC2 pore provides a suitable preparation for structural investigation of human TPCs, for which atomic information is currently lacking. Although its homomeric nature clearly distinguishes it from the wild- type pore (Fig. 1B), symmetrical pores are providing significant insight into the properties of their asymmetric counterparts. For example, prokaryotic Na_V_s are homotetramers that nonetheless bind and are regulated by the same Na^+^ channel antagonists that target asymmetric eukaryotic Na_V_^34^. Moreover, a mutant prokaryotic Na_V_, into which Ca^2+^ selectivity has been engineered (‘CavAb’) is targeted by eukaryotic Ca_V_ antagonists^35^. Our finding that Ca^2+^ uptake by the DI pore is sensitive to both Na^+^ and Ca^2+^ channel antagonists (Fig. 4B), similar to full-length TPCs^17^, suggests that essential pharmacological features are also preserved upon simplification of the TPC pore. In support, our docking analyses predict a drug binding site centred within the pore cavity comprising asparagine residues in S6 from multiple chains (N305 in full length TPC2) (Fig. 4I). This region corresponds to our previous *in silico* study using a wild-type TPC pore^17^ and the crystallographic mapping of a local anaesthetic within the pore of a prokaryotic Nav^34^. Notably, mutagenesis of conserved asparagines in domain I and III of eukaryotic Ns_V_ reduces sensitivity to local anaesthetics, suggesting a common binding site^36^,^37^. Thus, our reductionist approach might well be of relevance not only to two- but perhaps even four-domain channels, which currently represent a formidable challenge to structure-function analyses.

## Methods

### Bioinformatics

Multiple sequence alignments were carried out using ClustalOmega^38^. Sequences used, with accession/PDB codes in parentheses, were human TPC2 (AAH63008)^6^, Ns_V_Sp (AAR26729)^19^,^20^, Ns_V_Ba (AFV25941)^30^,^31^, Ns_V_Ae (4LTO)^20^, Ns_V_Ms (3ZJZ)^22^, Ns_V_Ab (4EKW)^39^, Ns_V_Rh (4DXW)^40^ and Ns_V_Ct (4BGN)^41^. Secondary structure predictions were performed using PSIPRED^42^ and JPred^43^. Disorder predictions were performed using PrDOS^44^, DisEMBL^45^ and DISOPRED^46^.

### Plasmids

For expression in HeLa cells, nucleotide sequences corresponding to the pore regions of TPC2 were amplified by PCR using IMAGE Clone 5214862 (BC063008) as the template and domain-specific primers (Table S1). The products were inserted in frame into EcoRI/XhoI sites of pCS2 + containing the coding sequence of GFP to generate C-terminally-tagged constructs. The DI pore mutant was generated by site-directed mutagenesis using primers previously used to introduce the L265P mutant in the full length channel^25^ (Table S1).

For expression in *E. coli* cells, nucleotide sequences corresponding to codon-optimised pore regions were synthesised by MWG Operon and inserted between the NdeI/BamHI sites of pET15b to generate N-terminally hexa-histidine-tagged constructs. The initial codon-optimised construct spanned S212-Q418 and incorporated the DI-DII linker. After initial expression trials (Fig. S1), a stop codon was introduced at the residue equivalent to A337 in the full length channel by site-directed mutagenesis using the primers described in Table S1.

DNA sequencing confirmed the correct sequences for all clones. pDsRed2-ER was obtained from Clontech Laboratories. The plasmid encoding LAMP1-mRFP was described previously^47^.

### Human cell culture and preparation of cell lysates

HeLa cells were cultured in DMEM Glutamax I (GIBCO) (a Ca^2+^-containing medium) supplemented with 10% fetal bovine serum (GIBCO), 100 U/ml penicillin and 100 μg/ml streptomycin (GIBCO) at 37 °C in a 5% CO_2_ humidified atmosphere. For transfection, cells were plated onto sterile 10 mm coverslips coated with 20 μg/ml poly-L lysine (Sigma) or 6-well tissue culture plates and transiently transfected with Lipofectamine^®^2000 (Invitrogen), as per the manufacturer’s protocol. Cell viability was reduced in cultures expressing either the DI and DII pores (data not shown).

To prepare lysates for Western blotting, cells were harvested by scraping, and then lysed in Ripa buffer (150 mM NaCl, 50 mM Tris, 0.5% (w/v) sodium deoxycholic acid, 0.1% (v/v) sodium dodecyl sulphate, 1% (v/v) Triton X-100, pH7.4), supplemented with EDTA-free protease inhibitors (Roche) and Halt^TM^ phosphatase inhibitor cocktail (Thermo Scientific) for 30 min on ice. Samples were centrifuged at 15,000 *g* at 4 °C for 15 min and the resulting supernatants stored at −20 °C. Protein concentrations were calculated using a bicinchoninic acid assay, calibrated to bovine serum albumin protein standards.

### Bacterial cell culture and protein purification

The DI pore constructs were overexpressed by the addition of 0.5 mM isopropylthiogalactoside to C41(DE3) *E. coli* cells grown in lysogeny broth containing 100 μg/ml ampicillin at an A_600_ of 0.7–0.9. Cells were cultured for a further 3 h at 37 °C or 18 hr at 22 °C (for expression trials), pelleted by centrifugation and stored at −80 °C. Cell pellets were resuspended at 4 ml/g of cells in lysis buffer (25 mM Tris, 300 mM NaCl, 0.2 mM phenylmethylsulfonyl fluoride (PMSF), 1 μg/ml DNAse I (Life Technologies), 2.5 mM MgSO_4_, pH7.4 supplemented with EDTA-free protease inhibitor (Roche)) and lysed by pressure homogenisation with an Avestin EmylsiFlex-C5. The resulting homogenate was centrifuged at 10000 *g* for 30 mins at 4 °C to pellet unlysed cells and inclusion bodies. The supernatant was then centrifuged at 200000 *g* for 2.5 hours at 4 °C to pellet the membrane fraction. The membrane pellets were resuspended in solubilisation buffer (25 mM Tris, 500 mM NaCl, 0.2 mM PMSF, pH7.4, supplemented with EDTA-free protease inhibitors and an excess of detergent) and incubated for 2 h at 4 °C. The detergents used (with % (w/v) used for solubilisation in parentheses; all from Anatrace) were: n-decyl β-D-maltopyranoside (DM, 0.87%) and in initial expression trials, lauryldimethylamine oxide (LDAO, 0.23%); n-dodecyl β-D-maltopyranoside (DDM, 1%) and Cymal-5 (1.2%). Unsolubilised material was removed by centrifugation at 20000 *g* for 30 mins at 4 °C. To bind His-tagged proteins, the soluble fraction was incubated with either Ni-NTA (Qiagen) or TALON (Clontech) affinity resin in the presence of 20 mM imidazole to reduce non-specific binding. After ~16 hours at 4 °C, the protein-bound resin bed was washed with 80x bed volume of buffer A (25 mM Tris, 500 mM NaCl, 0.174% (w/v) DM, pH7.4) containing 20 mM imidazole. Bound protein was eluted with 5x bed volume of buffer A containing 500 mM imidazole. The sample was then concentrated using a 50 kDa-cutoff Amicon Ultracentrifugal filter, diluted with buffer A and re-concentrated to remove imidazole.

For size exclusion chromatography, the concentrated sample (~100 μl) was loaded onto a Superdex 200 10/300 GL gel filtration column (GE Healthcare), connected to an AKTA FPLC and eluted with buffer A at 0.5 ml/min. The pureified protein (~300 μg from a 45 l culture) was concentrated to ~10 mg/ml, flash-frozen in liquid nitrogen and stored at −80 °C. Gel filtration columns were calibrated using protein standards (Sigma). The protein identity was confirmed by mass spectrometry at the Protein and Nucleic Acid Chemistry Facility, Cambridge University, UK.

The Ns_V_Ba pore (A138-Q265) was expressed and purified as previously described^31^.

### SDS PAGE and Western blotting

HeLa cell lysates (15 μg), raw and solubilised *E. coli* membranes, affinity and size exclusion chromatography fractions, or purified pore proteins (2.2 ng) were separated on NuPAGE Novex 4–12% BisTris SDS gels (Life Technologies) and where indicated, stained with Instant Blue coomassie stain (Expedeon).

For Western blotting, proteins were transferred to PVDF (BioRad or iBlot, Life Technologies) membranes according to standard procedures. The primary antibodies used were anti-GFP (rabbit polyclonal α-GFP, A11122 Life Technologies, 1 in 1000) and anti-TPC2 (rabbit polyclonal α-TPC2, Eurogentec custom antibody, 1 in 1000)^24^. Blots were developed using a secondary antibody (goat α-rabbit IgG/horseradish peroxidase (HRP) conjugate, 1706515 BioRad, 1 in 2000) and the ECL Prime Western Blotting System (GE Healthcare). All antibodies were incubated for 1 hr at room temperature. His-tagged proteins were detected using a monoclonal α-poly-histidine/alkaline phosphatase conjugate antibody (mouse, A5588 Sigma, 1 in 2000, 2 hrs at room temperature). Blots were developed using SIGMA*FAST* BCIP/NBT tablets (Sigma), as per the manufacturer’s instructions.

### Dynamic light scattering

Dynamic light scattering experiments were carried out using a pUNk instrument (Unchained Labs) according the manufacturer’s instructions. Purified protein (5 μl at 5 mg/ml) was loaded into a blade cell. Using an unbiased approach, the optimal traces from 10 runs were combined to calculate hydrodynamic diameters and polydispersity values.

### Synchrotron radiation circular dichroism (SRCD) spectroscopy

SRCD spectra were measured at the DISCO beamline at the Soleil Synchrotron (France). Data were collected in a 0.1 nm path-length demountable Suprasil cell (Hellman Ltd, UK) at 15 °C from 270 nm to 180 nm, using a 1 nm step size and a dwell time of 1.2 sec. Spectra (obtained in triplicate) were corrected for baselines using protein-free buffer, calibrated using camphor sulphonic acid, and scaled to units of delta epsilon using a mean residue weight value of 113.1. Processing was undertaken using the CDtools software^48^. Resulting spectra were analysed for secondary structure content using the DichroWeb analysis server^49^ with the CONTIN algorithm and the SMP180 reference dataset specifically designed for the analysis of membrane proteins^50^. Thermal melt experiments were performed between 15 °C and 95 °C by obtaining triplicate measurements at 222 nm, following 5 °C step increments and an equilibration period of 5 min.

### Confocal microscopy

Cells were fixed 4% (w/v) paraformaldehyde (VWR) for 10 min at room temperature and confocal images were captured using the LSM510 confocal scanner attached to an inverted Axiovert 200 M microscope (Zeiss) fitted with a Plan-Apochromat 63x water immersion objective. The excitation wavelengths (ex) and emission filters (em) were as follows: DAPI, ex 364 nm/em 385–470 nm; GFP, ex 488 nm/em 505–530 nm; RFP, ex 543 nm/em 560–615 nm. Zeiss ZEN2009 and ImageJ were used to acquire and present the images.

### Live cell Ca^2+^ imaging

Cytosolic Ca^2+^ concentration was measured using the ratiometric dye, Fura-2. HeLa cells were incubated for 1 hour with 2.5 μM Fura-2-AM (Life Technologies) and 0.005% (v/v) pluronic (Life Technologies) in HEPES-buffered saline (HBS; 1.25 mM KH_2_PO_4_, 2 mM CaCl_2_, 2 mM MgSO_4_, 3 mM KCl, 156 mM NaCl, 10 mM D-glucose and 10 mM NaHEPES, pH 7.4). Cells were washed in HBS and mounted in a 1 ml imaging chamber. Fluorescence images were captured with a cooled coupled device camera (TILL photonics) attached to an Olympus IX71 inverted fluorescence microscope, fitted with a 20x objective and a monochromator light source. Fura-2 was excited at 340/380 nm and emission captured using a 440 nm long-pass filter at 3 s intervals. Following base-line recording in HBS, cells were washed into Ca^2+^ free HBS (CaCl_2_ replaced with 1 mM EGTA) and stimulated with 1 μM thapsigargin (Merck). Transfected cells were identified prior to Ca^2+^ measurements by excitation at 488 nm, capturing emission with a 515 nm long-pass filter.

### Radiotracer uptake

Radiotracer uptake experiments were carried out based on previously validated protocols^19^,^51^. With this method, high concentrations of intraliposomal cations generate electrochemical gradients that drive extraliposomal radiotracer accumulation.

A 3:1 mixture of 1-palmitoyl-2-oleoyl-*sn*-glycero-3-phosphoethanolamine (POPE) and 1-palmitoyl-2-oleoyl-*sn*-glycero-3-phospho-(1′-*rac*-glycerol) (POPG, both from Avanti Polar Lipids) was sonicated in intraliposomal buffer supplemented with 37 mM CHAPS (Anatrace). The mixture was incubated at room temperature for 2 hours prior to addition of purified pore proteins (20 μg protein/mg lipid). Following a further 20 minute incubation, proteoliposomes were recovered by sequential centrifugation (1000 *g,* 20 s) through partially dehydrated Sephadex G50 columns (1 ml) pre-soaked in intra- and extra-liposomal buffer. The intraliposomal buffer was composed of 10 mM HEPES, 4 mM NMDG, 0.5 mM EGTA and either 300 mM BaCl_2_ (for ^45^Ca experiments) or 450 mM NaCl (for ^22^Na experiments). The extraliposomal buffers contained 400 mM D-sorbitol, 10 mM HEPES, 4 mM NMDG, with 0.5 mM EGTA for ^22^Na experiments only. All buffers were at pH7.4.

To initiate uptake, proteoliposomes were diluted into extraliposomal buffer supplemented with 2 μCi/ml ^45^Ca (specific activity: 25.6 mCi/mg) or 0.5 μCi/ml ^22^Na (specific activity: 633.8 mCi/mg) (both from Perkin Elmer). In some experiments, the extraliposomal buffer also contained either 100 μM diltiazem or 1 mM bupivacaine (both from Sigma). Unincorporated radiotracer was removed by passing triplicate samples (60 μl) at the stated time points, through NMDG-charged DOWEX cation exchange columns (0.5 ml) pre-soaked in 400 mM sorbitol (Sigma). Samples were eluted with 1 ml of 400 mM sorbitol and radioactivity determined by liquid scintillation counting. To reduce non-specific binding, the DOWEX columns were sequentially washed with 400 mM sorbitol containing 5 mg/ml BSA and then 10 mg/ml POPE/POPG prior to use.

### Homology modelling

Homology modelling of the human TPC2 DI pore monomer was carried out using the Phyre2 server^52^ with the crystal structure of AtTPC1 (pdb: 5E1J) as a template^12^, according to the alignment shown in Fig. S3. Residues D245-R251 in the putative turret loop between S5 and the first pore helix were excluded due to lack of suitable template. Energy minimisations were carried out with GalaxyRefine^53^, and the model with the lowest MolProbity score^54^ was assembled into a tetramer by structural alignment with AtTPC1, and further refined using GalaxyRefineComplex^53^. The pore interior cavity was visualised using HOLE^55^.

### Electrostatics calculations

Electrostatic potential energy calculations using the Poisson-Boltzmann equation were carried out using APBSmem^56^, as described previously^57^. The homology model of the DI pore was aligned along the *z*-axis and centred at 0,0,0. Partial charges and atomic radii were assigned to the homology model by PDB2PQR^58^ using the CHARMM parameter set^59^. A 300 × 300 × 300 Å^3^ map with 97 × 97 × 97 gridpoints was used within APBSmem. The 40 Å-thick hydrophobic membrane slab ranged between −25 Å and 15 Å in the *z*-axis, with a water-containing exclusion of 18 Å through the pore. The dielectric constants for water, membrane and protein were 80, 2 and 2, respectively, whilst the ionic strength was set at 0.1 M with columbic charges of ±1 and radius of 2 Å. The water probe radius was 1.4 Å, and the temperature was 298.15 K. The charge and surface models were Spl2 and Mol, respectively. Energy calculations were made for Ca^2+^, Na^+^ and Cl^−^ (with final Born radii of 1.73 Å, 1.68 Å and 1.88 Å, respectively) at 3 Å intervals along the *z*-axis.

### Docking

Structures of diltiazem and bupivacaine were downloaded from the ZINC database (http://zinc.docking.org/)^60^ and were docked to the structural model using Autodock4.2^61^ as previously described^17^,^62^. Docking was performed in a blind fashion using a 50 × 50 × 50 Å grid with 0.375 Å spacing. 100 docking runs were carried out using a Lamarkian Genetic Algorithm^63^ using default parameters. The top ranked pose for each compound was selected for presentation. Ligand-protein interactions were predicted using LigPlot^+^ (http://www.ebi.ac.uk/thornton-srv/software/LigPlus)^64^.

## Additional Information

**How to cite this article**: Penny, C. J. *et al*. Isolated pores dissected from human two-pore channel 2 are functional. *Sci. Rep.* **6**, 38426; doi: 10.1038/srep38426 (2016).

**Publisher's note:** Springer Nature remains neutral with regard to jurisdictional claims in published maps and institutional affiliations.

## Supplementary Material

Supplementary Data
