# Supplementary material for: Isolated pores dissected from human two-pore channel 2 are functional
Source: Sci Rep. 2016 Dec 12;6:38426. doi: 10.1038/srep38426 (PMC5150636; doi:10.1038/srep38426)
Supplement: Supplementary Data [file srep38426-s1.pdf]

Supplementary information

Isolated pores dissected from human two-pore channel 2 are functional.

Christopher J. Penny[1], Taufiq Rahman[2], Altin Sula[3], Andrew J. Miles[3], B. A. Wallace[3*] and Sandip Patel[1*]

[1]Department of Cell and Developmental Biology, University College London, London, WC1E 6BT, UK

[2]Department of Pharmacology, University of Cambridge, Cambridge, CB2 1PD, UK

[3]Institute of Structural and Molecular Biology, Birkbeck College, University of London, London, WC1E 7HX, UK

***Authors to whom correspondence should be addressed.**

Figure S1. Optimisation of TPC2 pore-only protein expression in *E. Coli*.

(**A-C**) Expression, solubilisation and purification trials of *E. coli* cells transformed with a plasmid encoding the hexa-histidine-tagged TPC2 DI pore-only construct containing an extended C-terminus (S212-Q418) (expected size: 20 kDa). (**A**) Western blot analysis using an α-poly-histidine antibody against membrane fractions isolated from *E. coli* cells during expression trials at 37ºC and 22ºC. (**B**) Western blot analysis using an α-poly-histidine antibody against membranes solubilised in 10x CMC of the detergents DM, Cymal5 and LDAO or in 1% (w/v) of DDM during solubilisation trials. (**C**) Coomassie-stained SDS PAGE analysis of the proteins eluted by 500 mM imidazole from washed Ni-NTA or TALON resin beads during affinity chromatography purification trials. (**D**) Expression trial of shortened TPC2 DI pore-only (S212-A337) (expected size: 14.6 kDa). This construct was used for all subsequent experiments.

Figure S2. Co-expression of TPC2 pores with a lysosomal marker

Confocal images of HeLa cells co-expressing a lysosomal marker (LAMP1-RFP, red) and the indicated GFP-tagged pore (green). Nuclei (blue) were stained with DAPI. Scale bar 10 µm. Colocalisation plots of red and green fluorescence intensity between arrowheads are shown below.

Figure S3. Homology model alignments

Alignments of human TPC2 (HsTPC2) and TPC1 from *Arabidopsis Thaliana* (AtTPC1) used for the generation of the homology model of the DI pore. For AtTPC1, transmembrane helices are highlighted in yellow and the re-entrant pore helices are highlighted in cyan. For HsTPC2, a short turret loop, denoted by ^ was not modelled.

Table S1. Primers

List of forward and reverse primers used to generate the pore-only constructs. Mutagenic bases are underlined. The *E. coli* primers target a codon-optimised plasmid.

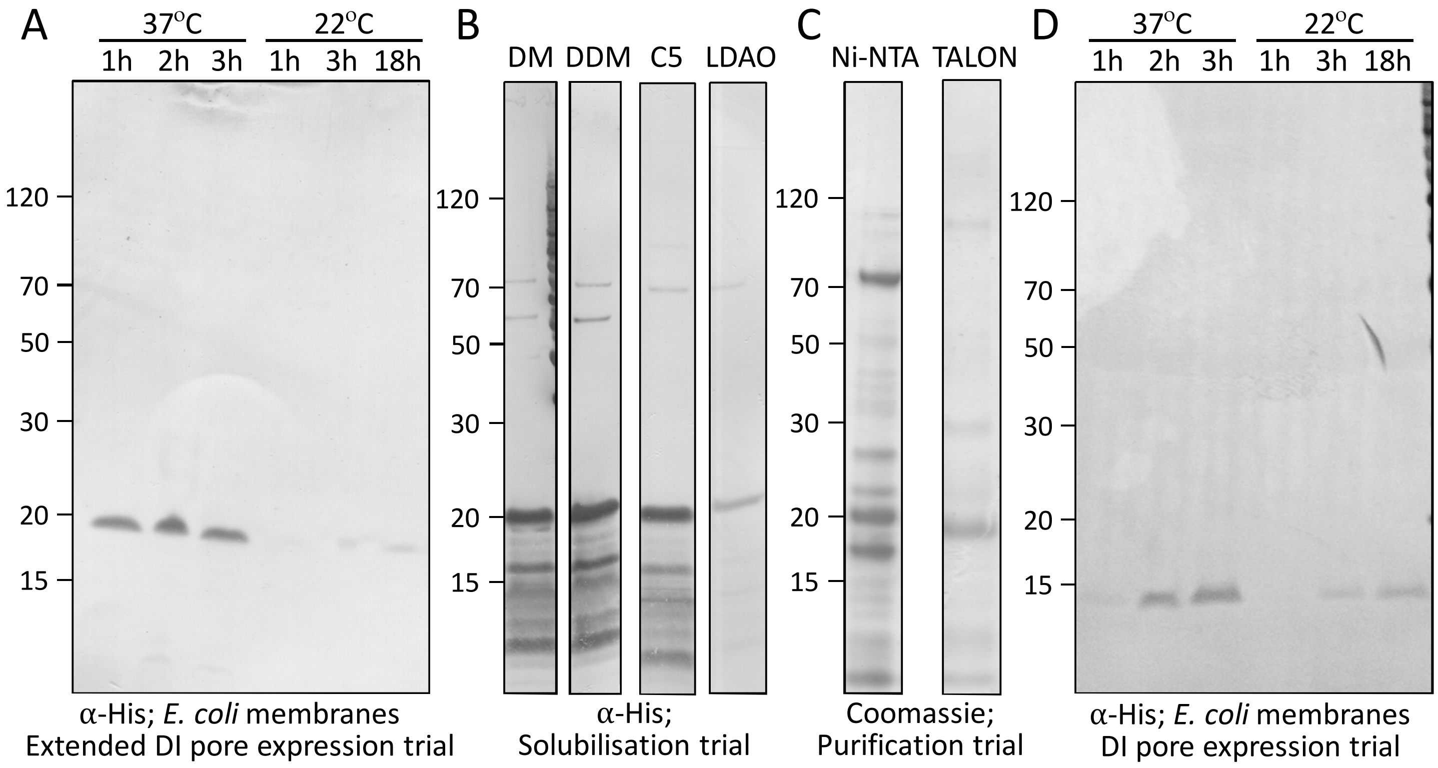
A
37°C
22°C
1h
2h
3h
1h
3h
18h
120
70
50
30
20
15
α-His; E. coli membranes
Extended DI pore expression trial
B
DM
DDM
C5
LDAO
120
70
50
30
20
15
α-His;
Solubilisation trial
C
Ni-NTA
TALON
120
70
50
30
20
15
Coomassie;
Purification trial
D
37°C
22°C
1h
2h
3h
1h
3h
18h
120
70
50
30
20
15
α-His; E. coli membranes
DI pore expression trial

Fig S1

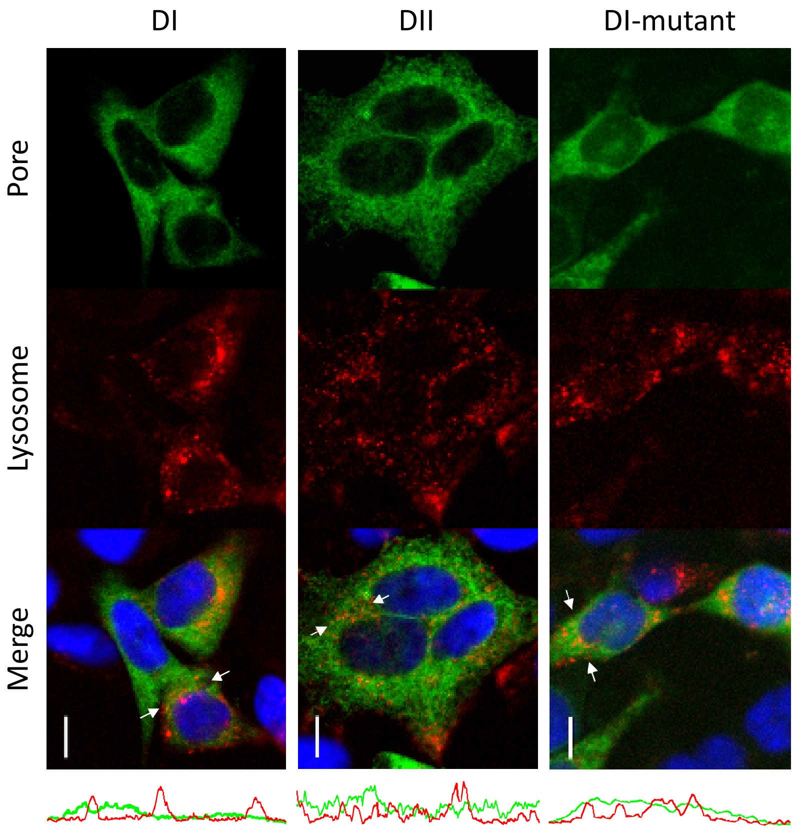

Fig S2

```
HsTPC2DI   SLPEMASVGLLLAIHLCLFTMFGMLLFAGGKQD^LTYFQNLPESLTSLLVLLTTANNPDV      277
            |    ::  |  : |   :    ::|   :|  || | :   :|  : :| ||:|||||
AtTPC1     MLGTYLNILALWMLFLLFASWIAFVMFEDTQQG^LTVFTSYGATLYQMFILFTTSNNPDV      270

HsTPC2DI   MIPAYSKNRAYAIFFIVFTVIGSLFLMNLLTAIIYSQFRGYLMKSLQTSLFRRRLGTRAA      337
            ||||  :|  ::||::: :||  |: ||: |::|  |:  | | :      :|     |
AtTPC1     WIPAYKSSRWSSVFFVLYVLIGVYFVTNLILAVVYDSFKEQLAKQVSGMDQMKRRMLEKA      330
```

Fig S3

**Cloning Primers**		
DI pore (HeLa)	Forward	5’-CACCGAATTCTGGATGGCCTCGCTGCCGGAAATG-3’
	Reverse	5’-AGGCTCGAGCATGGAGGATAGGACTTCAAAGG-3’
DII pore (HeLa)	Forward	5’-CACCGAATTCTGGATGGCCCTGGTGCAGAACATGCGT-3’
	Reverse	5’-AGGCTCGAGCCTGCACAGCCACAGGTG-3’
**Mutagenic Primers**		
DI-mutant (HeLa)	Forward	5'-GTCTCTGACTTCCCCCCTGGTGCTGCTGA-3'
	Reverse	5'-TCAGCAGCACCAGGGGGGAAGTCAGAGAC-3'
DI pore (*E Coli*)	Forward	5'-GTACTCGCGCAGCCTAGGAGGTTTGCGCTTGTG-3'
	Reverse	5'-CACAAGCGCAAACCTCCTAGGCTGCGCGAGTAC-3'

Table S1
